# Comment on: “Using Field Based Data to Model Sprint Track Cycling Performance”

**DOI:** 10.1186/s40798-021-00348-0

**Published:** 2021-08-23

**Authors:** Jamie Douglas

**Affiliations:** 1 High Performance Sport New Zealand (HPSNZ), Avantidrome, 15 Hanlin Road, Cambridge, 3283 New Zealand; 2 Cycling New Zealand, Cambridge, New Zealand

Dear Editor,

I commend Ferguson et al. [1] on elucidating the challenges faced by practitioners in optimising sprint cycling performance in their recent narrative review. Upon seeing the title, I was excited to read about field-based data and modelling of sprint cycling. However, I was surprised that the ‘data’ included only two power metre traces and that the authors performed no modelling. Furthermore, their recommendations were not supported by peer reviewed studies or anecdotal evidence.

They argue that peak power output (PPO) does not differentiate sprint race outcomes, contribute to power production over 15-60s and should not be an objective of training. This position is challenging to understand as perhaps the only study to have investigated the role of PPO per se in elite sprint cycling demonstrated a strong association between PPO (normalised to rider drag area) and flying 200 m velocity [2], which in turn largely predicts success in sprinting [3]. Furthermore, PPO has been demonstrated to explain ~74% of the variance in average power during a 30s Wingate test in trained athletes [4]. The authors argue that because a “substantial” proportion of adenosine triphosphate (ATP) regeneration is achieved via the oxidative pathway during efforts of 30-60s, sprint cyclists should instead prioritise ‘mixed power/endurance models’ of training. Whilst oxidative metabolism contributes 12-45% of energy resynthesis during efforts of 15-60s [5], metabolic energy release rates during sprint efforts of ≤ 60s are limited by mechanical demand and not rates of energy supply per se [6]. Therefore, maximal neuromuscular power (i.e. PPO) and the attenuation of fatigue-related impairments in contractile function (i.e. buffering) remain most critical to sustained maximal 15-60s power production during sprint cycling.

In an attempt to further downplay the importance of PPO, the authors present power-time/distance data from a 16-year-old female to suggest that sprint cycling is not an ‘all-out’ activity. However, deviations from an idiosyncratic power-duration relationship are typically observed during maximal sprint cycling due to fluctuations in resistance [7], variable pedalling rates [8] and the transition from standing to seated cycling (i.e. two distinct power-duration relationships [9]). These data likely represent ‘all-out’ effort following the initiation of the sprint (i.e. an optimal power-supply strategy for sprint cycling [10]).

Finally, whilst the sport of sprint cycling does involve multiple sprints, those usually occur with rest intervals of 40-120 min or longer [11]. In contrast, repeated sprint exercise (RSE) is defined as maximal efforts of ≤ 10 s interspersed with recovery periods of ≤ 60 s [12]. Consequently, findings on RSE are not applicable to the sport of sprint cycling. Indeed, evidence from elite BMX and Track Sprint athletes suggests no performance impairment across multiple bouts of simulated or actual competition [11, 13]. Thus, concerns about recovery between rounds raised by Ferguson et al. are not supported by previous literature.

In summary, oxidative metabolism contributes to sprint cycling performance and should be addressed with training, but not at the expense of PPO or maximal 15-60s power production. The recommendations provided in this narrative review are not evidence-based but represent unsubstantiated opinion and a selective interpretation of the literature.

## Data Availability

NA

## Abbreviations

ATP: Adenosine triphosphate
PPO: Peak power output
RSE: Repeated sprint exercise
